# BTG4 is A Novel p53 Target Gene That Inhibits Cell Growth and Induces Apoptosis

**DOI:** 10.3390/genes11020217

**Published:** 2020-02-19

**Authors:** Na Zhang, Tinghui Jiang, Yitao Wang, Lanyue Hu, Youquan Bu

**Affiliations:** 1 Department of Biochemistry and Molecular Biology, Chongqing Medical University, Chongqing 400016, China; zhangnayyl@163.com (N.Z.); jiangth@stu.cqmu.edu.cn (T.J.); 190372@cqmu.edu.cn (Y.W.); hulycqmu@163.com (L.H.); 2 Molecular Medicine and Cancer Research Center, Chongqing Medical University, Chongqing 400016, China

**Keywords:** BTG4, p53, promoter, proliferation, apoptosis

## Abstract

BTG4 is the last cloned and poorly studied member of BTG/Tob family. Studies have suggested that BTG4 is critical for the degradation of maternal mRNAs in mice during the process of maternal-to-zygotic transition, and downregulated in cancers, such as gastric cancer. However, the regulatory mechanism of BTG4 and its function in cancers remain elusive. In this study, we have for the first time identified the promoter region of the human BTG4 gene. Serial luciferase reporter assay demonstrated that the core promoter of BTG4 is mainly located within the 388 bp region near its transcription initiation site. Transcription factor binding site analysis revealed that the BTG4 promoter contains binding sites for canonical transcription factors, such as Sp1, whereas its first intron contains two overlapped consensus p53 binding sites. However, overexpression of Sp1 has negligible effects on BTG4 promoter activity, and site-directed mutagenesis assay further suggested that Sp1 is not a critical transcription factor for the transcriptional regulation of BTG4. Of note, luciferase assay revealed that one of the intronic p53 binding sites is highly responsive to p53. Both exogenous p53 overexpression and adriamycin-mediated endogenous p53 activation result in the transcriptional upregulation of BTG4. In addition, BTG4 is downregulated in lung and colorectal cancers, and overexpression of BTG4 inhibits cell growth and induces apoptosis in cancer cells. Taken together, our results strongly suggest that BTG4 is a novel p53-regulated gene and probably functions as a tumor suppressor in lung and colorectal cancers.

## 1. Introduction

The mammalian BTG (B-cell translocation gene)/Tob (Transducer of ErbB2) family comprises six members, including BTG1, BTG2/PC3/Tis21, BTG3/ANA, BTG4/PC3B, Tob1/Tob, and Tob2 [1,2]. Among the six members, BTG2, previously named as PC3 or Tis21, is the first to be identified independently by two groups [3,4]. So far, accumulating studies have demonstrated that the BTG/Tob family plays important roles in regulating various biological processes, including cell cycle progression, stress response, gene expression, and tumorigenesis [1,2]. 

The BTG/Tob proteins are structurally characterized by the highly conserved N-terminal BTG domain, which contains two conserved motifs, BOX-A and BOX-B, whereas their C-terminal regions are less conserved. Biochemical data revealed that the conserved BTG domain prominently function as a protein–protein interaction module. It has been reported that the BTG domain can interact with several DNA-binding transcription factors to mediate its role in transcriptional regulation. For example, BTG1 and BTG2 can interact with Hoxb9 [5], BTG3 interacts with E2F1 [6], and BTG1 can interact with the nuclear receptor TRa and the myogenic factor MyoD [7]. The BTG domain is also capable of interacting with CNOT7 and CNOT8, two deadenylase subunits of the Ccr4-Not complex involved in regulating mRNA deadenylation and turnover [8,9,10]. 

Studies suggest that BTG/Tob proteins play important roles in tumorigenesis and cancer. Suzuki et al. reported that Tob1 inhibits cell growth by decreasing cyclin D1 expression, and phosphorylation-mediated inactivation of Tob1 by Erk1 and Erk2 is required for Ras-mediated cell proliferation and transformation [11]. Mice lacking Tob1 are prone to spontaneous carcinogenesis with high frequencies of tumors [12]. BTG1 and BTG2 have been found to be frequently downregulated or mutated in several types of cancers, and the decreased expression of BTG1 and BTG2 is positively associated with malignant cell behavior and poor outcome in cancer patients [2,13]. BTG3 has also been shown to be downregulated in several cancers, and its expression might predict survival and prognosis in cancer patients [14,15,16]. Mice lacking BTG3 also display an increased incidence of lung tumors [1,14].

BTG4 is the last cloned BTG/Tob family member, and thus remains poorly studied [17]. Two recent studies reported that BTG4 is highly expressed and critical to degrade maternal mRNAs in mice during the highly conserved process of maternal-to-zygotic transition (MZT) [18,19,20]. BTG4 fosters maternal RNA degradation by interacting with CNOT7 and CNOT8, two components of the RNA de-adenylation complex CCR4-NOT. Three studies also suggested that BTG4 is downregulated in gastric cancer, chronic lymphocytic leukemia, and colorectal cancer [21,22,23]. Those studies highlighted the physiological and pathological significances of BTG4 expression. However, the regulatory mechanism of BTG4 still remains elusive, which needs further exploration. In the present study, we have for the first time cloned and identified the promoter region of the human BTG4 gene. We also found that BTG4 is a p53-regulated gene and probably functions as a tumor suppressor, at least in lung and colorectal cancers. 

## 2. Materials and Methods

### 2.1. Cell Culture

Human lung cancer cells H1299 and A549 and colon cancer cells HCT116 were obtained from Cell Resource Center, Shanghai Institutes for Biological Science, Chinese Academy of Sciences. H1299 were cultured in RPMI 1640 (Hyclone), and A549 were cultured in DME-F12 (Hyclone), and HCT116 were cultured in DMEM (Hyclone) supplemented with 50 units/mL penicillin, 50 mg/mL streptomycin, and 10% (v/v) FBS (Invitrogen). These cells were cultured in a 37 °C incubator in a humidified atmosphere containing 5% CO_2_. 

### 2.2. Cloning of BTG4 Gene Promoter Region

The luciferase reporters BTG4-P1837 (−1611/+226), BTG4-P647 (−421/+226), BTG4-P412 (−421/−9), and BTG4-p388 (−397/−9) were established by cloning the BTG4 promoter region into pGL3-basic vector using a seamless cloning kit (Novorec PCR NR001, Novoprotein, Shanghai, China). The primer sequences as well as restriction enzymes are provided in Table 1. The cloned fragments were verified by direct DNA sequencing. 

### 2.3. Site-Directed Mutagenesis

The luciferase reporters P412-M1, P412-M2, P412-M3, and P412-M4 were constructed by a site-directed mutagenesis kit (TOYOBO, Osaka, Japan) based on the parental construct BTG4-P412 (-421/-9) according to the manufacturer’s instruction. For M1 mutant, the putative Sp1 binding site of GGGCGA at − 115 bp was mutated to TCTACT. For M2 mutant, the putative Sp1 binding site of GGGGCG at −80 bp was mutated to GTTGAG. For M3 mutant, the putative Sp1 binding site of GGGGCG at −75 bp was mutated to GCTATG. For M4 mutant, the putative Sp1 binding site of GGGAGG at −48 bp was mutated to GTTCGG. All the mutations were confirmed by DNA sequencing. The primer sequences are provided in Table 1. 

### 2.4. Transfection and Luciferase Reporter Assay

For the luciferase reporter assays, cells were seeded in 12-well plates in triplicate and then co-transfected with the indicated plasmids using Neofect DNA^®^ transfection reagent (Neofect, Beijing, China). The pRL-TK vector (Promega, Madison, America) encoding Renilla luciferase was used as an internal control to monitor the transfection efficiency. Forty-eight hours after transfection, cells were lysed, and luciferase activity was determined using the Dual-Luciferase assay system (Promega, Madison, America) as described previously [24,25].

### 2.5. DNA Sequence Alignment and Database Mining 

The BTG4 mRNA and promoter sequences were acquired from the NCBI and UCSC database (https://genome.ucsc.edu/). Transcription factor binding sites were analyzed by online software MatInspector. Alignment for the BTG4 gene promoters from the human, mouse, and rat were carried out by online software Clustal Omega (https://www.ebi.ac.uk/Tools/msa/clustalo/). 

### 2.6. RNA Extraction and Quantitative Real-Time RT-PCR 

Total RNA was extracted from cells using TRIzol (Sigma, Tokyo, Japan) according to the manufacturer’s instruction. cDNA was synthesized from 500 ng of total RNA using a PrimeScript™ RT Master Mix kit (TaKaRa, Daliang, China). Real-time PCR was conducted by using the SYBR Premix Ex Taq (Perfect Real Time, TAKARA) as described previously [2,26]. The sequences of the primers were Forward: 5’-AGAAGTCACTGGCACTCTGA-3’ and Reverse: 5’-CCATTCCTCCCATCTGCCTT-3’ for BTG4, and Forward: 5’-ACCTGACCTGCCGTCTAGAA-3’ and Reverse: 5’-TCCACCACCCTGTTGCTGTA-3’ for GAPDH as an internal control for quantitative RT-PCR. 

### 2.7. Cell Proliferation Assay 

HCT116 cells were transfected with empty vector (pcDNA3.0-Flag) and BTG4 expression plasmid (pcDNA3.0-Flag-BTG4). Forty-eight hours later, cells were seeded into 96-well plates at a density of 3000 cells per well. The CCK8 reagent was added at 0, 24, 48, 72, and 96 h after seeding and incubated for 1 h at 37 °C. The resultant optical densities (ODs) were measured at 450 nm. 

### 2.8. Wound-Healing Assay 

H1299 cells were transfected with empty vector (pcDNA3.0-Flag) and BTG4 expression plasmid (pcDNA3.0-Flag-BTG4). Forty-eight hours later, cells were scratched with a 10-µL pipette tip, and washed twice with PBS, and finally incubated in serum-free RPMI-1640. Images were taken at 0 and 24 h with a Leica light microscope (DM4B, Leica Corporation, Wetzlar, Germany). The migration rate was quantified by calculating the wound area changes at the indicated time points. 

### 2.9. Apoptosis Assay 

An annexin V-fluorescein isothiocyanate (FITC) apoptosis detection kit (BD Pharmingen, Franklin lake, America) was used to determine apoptosis according to the manufacturer’s instructions. In brief, cells were collected, washed twice in ice-cold 1× PBS, and resuspended in binding buffer at a concentration of 1 × 10^6^ cells/mL. Annexin V-FITC and propidium iodide (PI) were then added and incubated for 15 min at room temperature in the dark. Cells were finally analyzed for apoptosis by flow cytometry. 

## 3. Results

### 3.1. Gene Organization and Chromatin State of the BTG4 Gene Locus 

**Figure 1:**
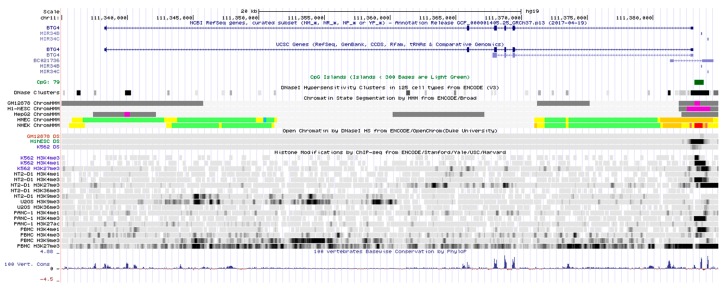
BTG4 gene structure and chromosomal state. The human genomic region harboring the BTG4 gene (chr11:111,3327,050-111,394,285 67,236 bp, human species genomic assembly version, GRCh37/hg19) is schematically represented with the indicated tracks (http//genome.ucsc.edu/). As for the chromatin state segmentation, the active promoter is shown in bright red, inactivated/poised promoter in purple, strong enhancer in orange, insulator in blue, transcriptional elongation in deep green, and weakly transcribed region in light green.

To better understand the genomic organization and chromatin state of the human BTG4 gene, we retrieved the UCSC genome browser (http://genome.ucsc.edu/). As shown in Figure 1, the BTG4 gene is mapped at chromosome11q23.1 and comprises six exons and five introns. A classical CpG island exists immediately upstream of the BTG4 transcription start site. The CpG island overlaps with regions marked by DNase I hypersensitivity and enriched with H3K4me3 marks (a hallmark of transcriptional initiation), suggesting that the CpG island harbors the promoter of the BTG4 gene. Interestingly, two human miRNA genes of miR-34b and miR-34c are located upstream of BTG4 and oriented in a head-to-head manner, implying that the BTG4 promoter might function as a bidirectional promoter. 

### 3.2. Identification of the BTG4 Promoter Region 

**Figure 2:**
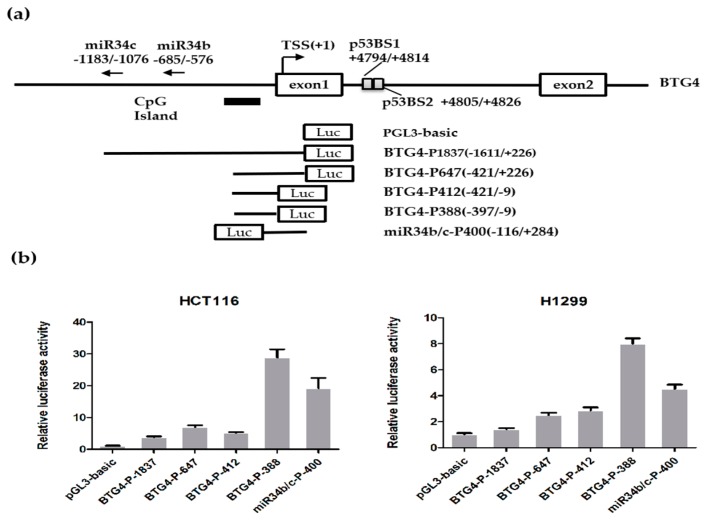
Identification of the BTG4 promoter region. (**a**) Schematic diagram of the BTG4 gene promoter reporter constructs. The positions relative to the major BTG4 transcription start site (+1) are indicated. The first and second exons of BTG4 are displayed by two open boxes. The two overlapped p3 binding sites (p53BS1/2) are shown by gray boxes. MiR34b and miR34c are transcribed in the opposite orientation from BTG4. The CpG island is indicated by thick black lines. (**b**) Luciferase assay. HCT116 and H1299 cells were transiently co-transfected with the indicated luciferase reporter constructs together with pRL-TK. Forty-eight hours later, luciferase activities were determined by the Dual Luciferase Assay System (Promega). Data are shown as the fold induction compared to that of the empty pGL3-basic vector. The results are presented as the mean and standard deviation (S.D.) of triplicates from a representative experiment.

To identify the potential proximal promoter region of the human BTG4 gene, a series of luciferase reporters, including BTG4-P1837(−1611/+226), BTG4-P647(−421/+226), BTG4-P412(−421/−9), and BTG4-P388(−397/−9), were established by cloning the different sizes of 5′-flanking genomic fragments of the BTG4 gene into a pGL-3-basic promoter-less vector (Figure 2a). As displayed in Figure 2b, the luciferase reporter assay showed that the luciferase activities of all four aforementioned constructs are significantly enhanced when compared to pGL3-basic in both HCT116 and H1299 cells. Among the four constructs, the activity of BTG4-P388(−397/−9) is the highest while that of BTG4-P1837(−1611/+226), BTG4-P647(−421/+226), and BTG4-P412(−421/−9) are relatively lower in both HCT116 and H1299 cells, suggesting that the core promoter region of the BTG4 gene is mainly located in the 5′-flanking 412-bp region corresponding to the BTG4-P412(−421/−9) construct. Intriguingly, the shortest construct BTG4-P388(−397/−9) showed much higher promoter activity in both cell lines than that of BTG4-P412(−421/−9), which contains the promoter fragment only 24 bp longer than that of BTG4-P388(−397/−9). This result strongly suggests that the 24-bp fragment from −421 to −397 bp might contain a strong silencer element. 

As the gene organization analysis revealed that BTG4 and its adjacent miR-34b/c are oriented head to head, we established an overlapped promoter reporter for miR-34b/c to determine whether the intergenic region has bidirectional promoter activity. As shown in Figure 2b, miR-34b/c-P400 shows remarkable promoter activity comparable to that of the shortest construct BTG4-P388 (−397/−9). This intriguing result suggests that BTG4 and miR-34b/c might share an intergenic bidirectional promoter. 

### 3.3. Sequence Analysis of the BTG4 Promoter Region 

**Figure 3:**
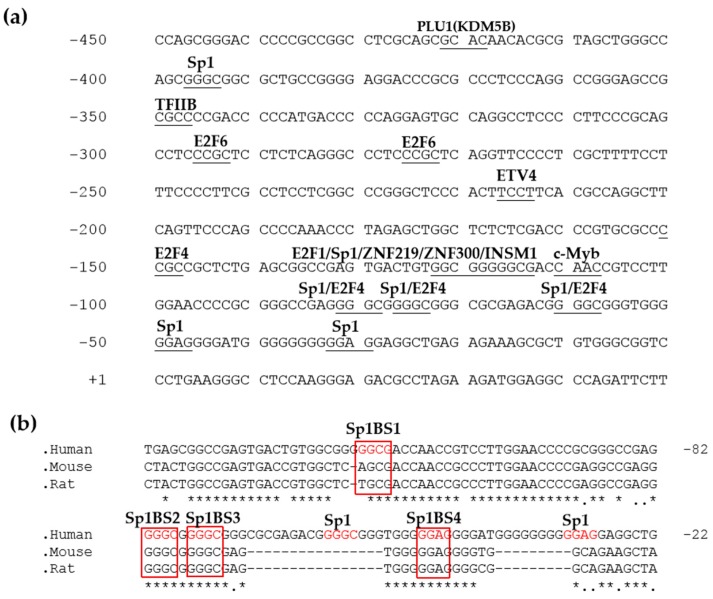
Nucleotide sequences of the human BTG4 gene promoter. (**a**) The putative transcription factor binding sites in the promoter region of the BTG4 gene were predicted and underlined. Nucleotides are numbered based on the major transcription start site of BTG4 (+1). (**b**) Conservative analysis of the BTG4 promoter sequence. Sequence alignment of the nucleotide sequences of partial BTG4 gene promoters from the indicated species was conducted by the online software Clustal Omega. The putative Sp1-binding sites are boxed. Identical bases among different species are marked with stars (*).

To understand the transcriptional regulatory mechanism of the human BTG4 gene, we used the online software MatInspector to predict potential transcriptional factor binding sites in the promoter region of the BTG4 gene. As displayed in Figure 3, the BTG4 gene lacks the classical TATA box but contains potential transcription factor binding sites, such as Sp1, E2F4, etc. (Figure 3a). Multiple sequence alignment analysis revealed that the four Sp1 binding sites are evolutionarily conserved across several species, including the human, mouse, and rat (Figure 3b), suggesting that Sp1 might play an important role in regulating the transcription of the human BTG4 gene. 

### 3.4. Functional Analysis of Sp1 Binding Sites in the BTG4 Gene Promoter 

**Figure 4:**
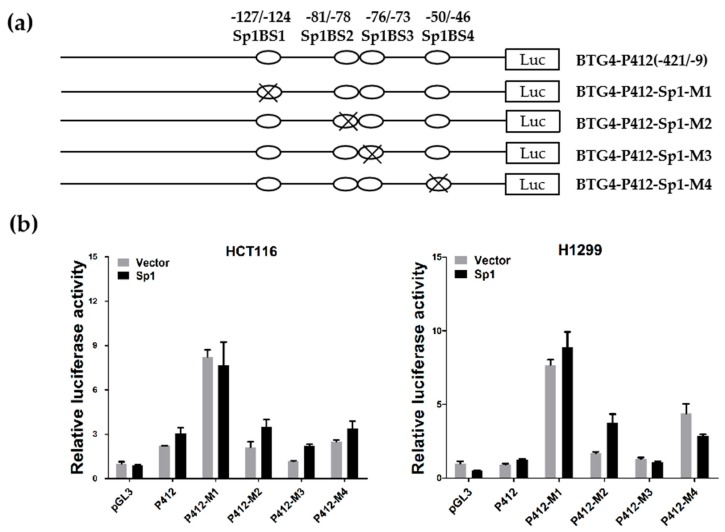
Functional analysis of Sp1 binding sites in the BTG4 promoter. (**a**) Schematic diagram of site-directed mutagenesis of Sp1 binding sites in the BTG4-P412 luciferase reporter harboring the BTG4 promoter. The four potential Sp1 binding sites are indicated as open boxes (Sp1BS1, Sp1BS2, Sp1BS3, and Sp1BS4). The indicated point mutations are denoted by a cross at the potential Sp1 binding sites. (**b**) Luciferase reporter assays. The indicated luciferase reporter constructs were transfected into HCT116 and H1299 cells together with empty vector or with Sp1 expression plasmid. Forty-eight hours later, their luciferase activities were determined, as in Figure 3d.

As Sp1 binding sites are prominently enriched in the proximal promoter of the BTG4 gene, we decided to further verify their roles in BTG4 transcription regulation. To this end, wildtype BTG4-P412 was used as a template to establish four mutant reporter constructs, P412-Sp1-M1, P412-Sp1-M2, P412-Sp1-M3, and P412-Sp1-M4, in which the four evolutionally conserved putative Sp1 binding sites in the BTG4 proximal promoter region were disrupted by site-directed point mutation, respectively (Figure 3b and Figure 4a). As shown in Figure 4b, disruption of each of the four Sp1 binding sites did not attenuate the constitutive promoter activity of the BTG4 gene in both HCT116 and H1299 cells. Intriguingly, disruption of Sp1BS1 even caused a remarkable increase in the promoter activity of the BTG4-P412 reporter in both HCT116 and H1299 cells, suggesting that Sp1BS1 might function as a potential silencer. In addition, exogenous overexpression of Sp1 had negligible effects on the promoter activity of wildtype BTG4-P412 as well as most of the four mutant reporter constructs, P412-Sp1-M1, P412-Sp1-M2, P412-Sp1-M3, and P412-Sp1-M4. These results together strongly suggest that Sp1 is not a critical transcription factor for the transcriptional regulation of BTG4. 

### 3.5. Identification of a p53-Responsive Element with the First Intron of the BTG4 Gene 

**Figure 5:**
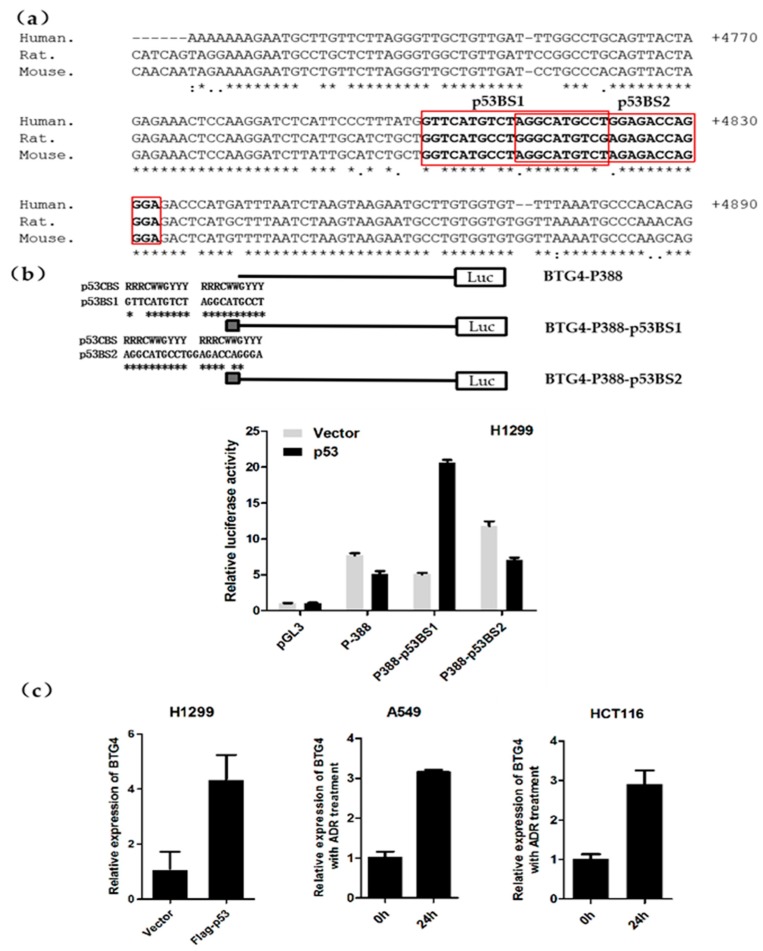
BTG4 gene contains a p53 response element and is induced by p53. (**a**) Partial sequences of the first intron of the BTG4 gene were aligned across the human, mouse, and rat by online software Clustal Omega. The position is relative to the transcription start site of BTG4. The two overlapped p53 binding sites are indicated as p53BS1 and p53BS2. (**b**) The two overlapped p53 binding sites were cloned into the wildtype reporter BTG4-P388, respectively. The indicated reporters were cotransfected into H1299 cells with empty or Flag-p53 expression plasmids and pRL-TK. The luciferase activities were determined as described in Figure 2. The two potential p53 binding sites of p53BS1 and p53BS2 compared to the consensus p53 binding site (p53 CBS). R, purine; Y, pyrimidine; W, adenine or thymine. (**c**) H1299 cells were transiently transfected with empty vector (pcDNA3.0-Flag) or pcDNA3.0-Flag-p53 expression plasmid. Forty-eight hours later, cells were collected and subjected to RT-PCR analysis. A549 and HCT116 cells were treated with adriamycin at the final concentration of 1μM. Cells were then collected and subjected to RT-PCR analysis at the indicated time points.

To further explore the transcription factors that regulate BTG4 gene expression, we retrieved the transcription factor binding sites in the whole genomic sequences of the BTG4 gene. The analysis revealed that the first intron of the BTG4 gene contains two overlapped p53 binding sites, namely p53BS1 and p53BS2 (Figure 2a and Figure 5a). Of note, the two p53 binding sites are highly conserved among the human, mouse, and rat (Figure 5a). To determine whether the two p53 binding sites are responsive to p53 expression, the two sites were cloned into the BTG4-P388 reporter to establish the BTG4-P388-p53BS1 and BTG4-P388-p53BS2 constructs (Figure 5b). The luciferase assay revealed that exogenous overexpression of p53 significantly enhanced the promoter activity of BTG4-P388-p53BS1 but not BTG4-P388-p53BS2, suggesting that only the first p53 binding site is responsive to p53. 

### 3.6. P53 Induces BTG4 Transcription 

Next, we decided to determine whether BTG4 transcription can be regulated by p53. As shown in Figure 5c, exogenous overexpression of p53 caused a significant increase of BTG4 expression at the mRNA level in H1299 cells. In support of this, BTG4 transcription was also induced by adriamycin treatment in A549 and HCT116 cells. Adriamycin is a chemotherapeutic agent, which is known to be able to cause endogenous activation of p53. Therefore, the results suggest that p53 induces BTG4 transcription. In addition, our preliminary experiment using HCT116 wildtype and p53-/- cells also suggested that the transcription of BTG4 was induced in a manner dependent on the presence of p53 (Supplementary Figure S1). 

### 3.7. BTG4 Inhibits Cell Proliferation and Migration, and Induces Apoptosis 

**Figure 6:**
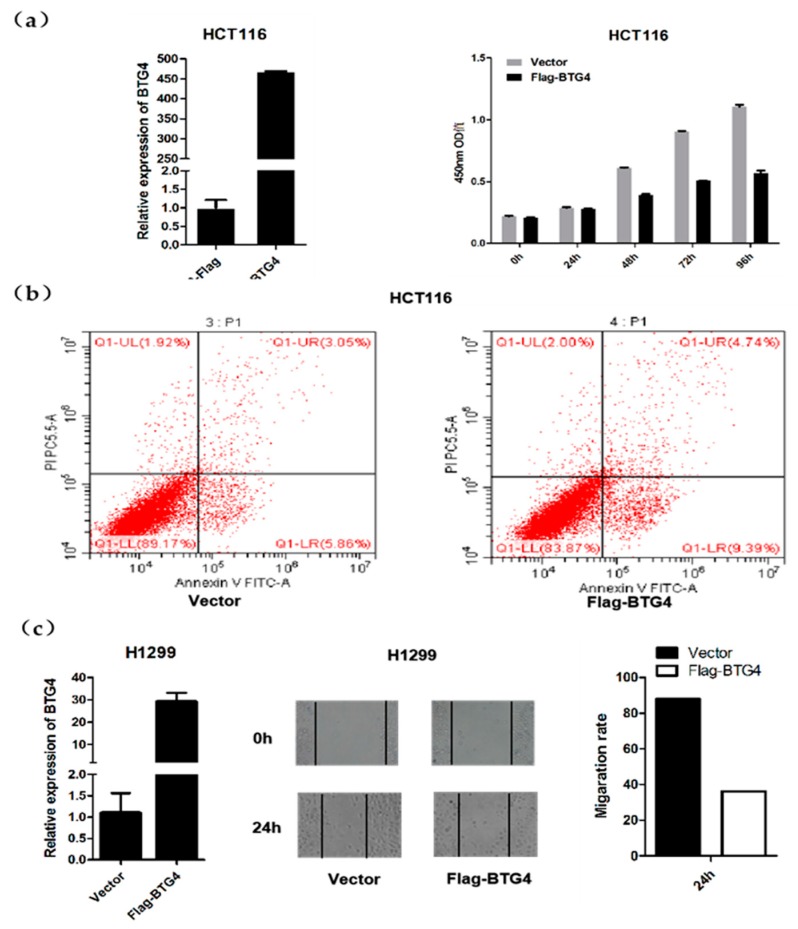
BTG4 inhibits cell proliferation and migration, and induces apoptosis. (**a**) HCT116 cells were transiently transfected with empty vector (pcDNA3.0-Flag) or pcDNA3.0-Flag-BTG4 expression plasmid, and then subjected to cell proliferation assay. (**b**) HCT116 cells were transfected as in (a). Forty-eight hours later, cells were harvested and subjected to apoptosis assay. The lower-left quadrant represents viable cells (FITC^−^/PI^−^); lower right represents early apoptotic cells (FITC^+^/PI^−^); upper right represents late apoptotic and secondary necrotic cells (FITC^+^/PI^+^). (**c**) H1299 cells were transiently transfected with empty vector (pcDNA3.0-Flag) or pcDNA3.0-Flag-BTG4 expression plasmid, and then subjected to the wound healing assay. Cell migration status was monitored under a microscope at the indicated time points, and subsequently quantified.

We then determined whether BTG4 could affect cancer cell proliferation, migration, and apoptosis. To this end, HCT116 as well as H1299 cells were transiently transfected with BTG4 expression plasmid. As shown in Figure 6a, exogenous overexpression of BTG4 significantly inhibited cell proliferation in HCT116 cells. Annexin V-FITC and PI double staining with flow cytometry was then conducted to determine apoptotic cells. As shown in Figure 6b, approximately 9.39% and 4.74% of BTG4-overexpressed cells displayed an early apoptotic (FITC^+^/PI^−^) and late apoptotic/secondary necrotic phenotype 48 hours after transfection, whereas only 5.86% and 3.05% of control cells showed this phenotype. As shown in Figure 6c, exogenous overexpression of BTG4 remarkably suppressed migration in H1299 cells as detected by the wound healing assay. Of note, as the BTG/Tob family has been shown to play important roles in regulating cell cycle progression [1,2], the proliferation inhibition and apoptosis induction caused by BTG4 overexpression is probably preceded by potential cell cycle arrest. 

### 3.8. The Expression of BTG4 in Normal and Cancer Tissue

**Figure 7:**
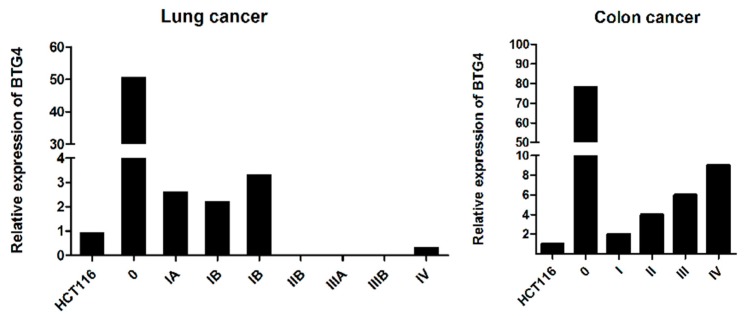
The expression of BTG4 mRNA in lung and colon cancers. BTG4 expression levels in normal and cancerous tissues were analyzed with quantitative RT-PCR using OriGene TissueScan cancer panels (CSRT101). 0 denotes normal lung or colorectal tissues. I, II, III, IV represent different clinical stages of lung and colorectal cancers.

Finally, we examined whether the expression of BTG4 is aberrantly changed in cancer tissues. As shown in Figure 7, BTG4 mRNA was significantly decreased in both lung and colon cancer samples compared with their normal counterpart tissues. Of note, in lung cancer tissues, the expression levels of BTG4 mRNA were significantly associated with the clinical stage. 

## 4. Discussion

In the present study, we firstly identified the promoter region of the human BTG4 gene, which might not be mainly regulated by Sp1 although it contains several GC boxes. Our results strongly suggest that BTG4 is a novel target gene of p53 as evidenced by the fact that its first intron contains a canonical p53 response element and its transcription could be induced by p53. As a well-known tumor suppressor, p53 mainly functions as transcription factor to regulate the expression of various downstream target genes, consequently participating in diverse biological processes, including DNA damage response, cell cycle progression, apoptosis, autophagy, metabolism, etc. [27,28,29]. Thus, identification of novel p53-target genes would provide greater insight into the molecular mechanisms that govern the tumor suppressor functions of p53. Previously reported well-known p53 targets include Bax and PUMA involved in apoptosis, CDKN1A in cell cycle arrest, TIGAR in the metabolism of glycolysis, and DRAM in autophagy [27]. It has been reported that both BTG2 and BTG3 are directly regulated by p53 and implicated in the DNA damage response pathway as well as apoptosis [6,30]. As our results demonstrated that BTG4 is induced upon the treatment of adriamycin, which could cause DNA double breaks, it is of interest to further investigate the role of BTG4 in the DNA damage response pathway. 

Our data also found that the expression of BTG4 is downregulated in lung and colon cancers, and overexpression of BTG4 can inhibit proliferation and migration, and induce apoptosis, suggesting that similar to BTG2 and BTG3, BTG4 might also act as a potential tumor suppressor. Our results are consistent with the previous report by Toyota et al., who found the 5′-flanking region of BTG4 is hypermethylated and overexpression of BTG4 inhibits the colony formation ability in colorectal cancer cells [23]. Although Ou et al. reported that BTG3 binds to and inhibits E2F1 through its conserved N-terminal BTG domain, our preliminary results revealed that BTG4 does not bind to E2F1 in a similar manner. As the recent studies have demonstrated that BTG4 could interact with CNOT7 and CNOT8 to foster maternal mRNA degradation during maternal-to-zygotic transition [18,19,20], further study is needed to investigate whether BTG4 regulates mRNA deadenylation through a similar mechanism in tumorigenesis. Of note, Liu et al. and Yu et al. also revealed that BTG4 transcripts are highly enriched in the ovary and testis rather than somatic tissues. We also found that BTG4 is expressed at a very low level in somatic cells and its expression is difficult to detected by routine immunoblotting. Thus, highly sensitive methods for detecting BTG4 protein expression need to be developed in the future. However, the current data do not rule out the possibility that the BTG4 gene might execute its functions only through its RNA transcripts rather than proteins. 

Of note, two microRNAs miR34b and miR34c exist upstream of the BTG4 gene (Figure 2). Our results suggested that BTG4 and miR34b/c share a bidirectional promoter and constitute a head-to-hear gene pair [24] (Figure 3). Interestingly, previous reports showed that miR34b/c is directly regulated by p53 and implicated in cancer development and apoptosis [31]. Thus, the BTG4 and miR34b/c gene pair could be coordinately regulated by p53 as well as other transcription factors. Our results suggested that the 24-bp region from −421 to −397 bp might contain a strong silencer element (Figure 2a,b). Transcription factor binding analysis revealed that this 24-bp region contains a high-scored cis-acting element for PLU1 or JARID1B, now officially named lysine demethylase 5B (KDM5B). KDM5B is a lysine-specific histone demethylase that belongs to the jumonji/ARID domain-containing family of histone demethylases. It is capable of demethylating tri-, di-, and monomethylated lysine 4 of histone H3. Studies showed that KDM5B is upregulated in certain cancers and plays roles in the transcriptional repression of tumor suppressor genes, such as BRCA1 [32,33]. Thus, we speculated that KDM5B could be a transcriptional repressor for the BTG4 gene. Mutation of the first Sp1 binding site at −113 to −118 bp (BTG4-P412-Sp1-M1) also caused remarkably increased promoter activity compared with the wildtype reporter BTG4-P412, strongly suggesting a robust silencer(s) exists within or overlaps with this Sp1 binding site. Transcription factor binding analysis indicated that the first Sp1 binding site also serves as a high-scored cis-acting element for transcriptional repressors, such as ZNF219, ZNF300, and INSM1 [34,35,36,37]. Of note, INSM1 (insulinoma-associated 1) has been shown to be implicated in some cancers, and function as a transcriptional repressor [34,35,36,37]. Collectively, future works are needed to investigate whether these transcriptional repressors, especially KDM5B and INSM1, regulate the transcription of BTG4 as well as miR34b/c.

## 5. Conclusions

Human BTG4 gene contains several potential Sp1 and E2F binding sites in its promoter region, and one functional p53 consensus binding site in its first intron. Sp1 might not directly regulate BTG4 transcription. Overexpression of BTG4 inhibits cell growth and induces apoptosis in cancer cells. BTG4 is downregulated in lung and colorectal cancers. BTG4 is a novel p53-regulated gene and probably functions as a tumor suppressor in lung and colorectal cancers. 

## Figures and Tables

**Table 1:** The primer sequences for BTG4 promoter reporter construction.

Constructs	Primer Sequences and Templates
BTG4-P1837(−1611/+226)	F:5′-GCTAGCCCGGGCTCGAGATCTACTGTGGCAGGAACTGAGATGGA-3′ *Bgl* II R:5′-CAGTACCGGAATGCCAAGCTTGCTACCCAAGCCCACCTCCATTT-3′ *Hin*d III PCR Template: human genomic DNA
BTG4-P647(−421/+226)	F:5′-GCACAACACGCGTAGCTGGGCCA-3′ R:5′-GGTACCTATCGATAGAGAAATGTTCTGGC-3′ *Kpn* I PCR Template: BTG4-P1837
BTG4-P412 (−421/−9)	F:5′-AAGCTTGGCATTCCGGTACTGTTGGTAA-3′ *Hin*d III R:5′-CAGCGCTTTCTCTCAGCCTCCTCC-3′ PCR Template: BTG4-P647
BTG4-P388(−397/−9)	F:5′-GGGCGGCGCTGCCGGGGAGGA-3′ R:5′-GGTACCTATCGATAGAGAAATGTTCTGGC-3′ *Kpn* I PCR Template: BTG4-P412
BTG4-P388-p53BS2	F:5′-AGGCATGCCTGGAGACCAGGGAGCACAACACGCGTAGCTGGGCCA-3′ R:5′-GGTACCTATCGATAGAGAAATGTTCTGGC-3′ *Kpn* I PCR Template: BTG4-P388
BTG4-P388-p53BS1	F:5′-GTTCATGTCTAGGCATGCCTGGGCGGCGCTGCCGGGGAGGA-3′ R:5′-GGTACCTATCGATAGAGAAATGTTCTGGC-3′ *Kpn* I PCR Template: BTG4-P388
BTG4-P412-Sp1-M1	F:5′-TCTACTCCAACCGTCCTTGGAACCC-3′ R:5′-CCGCCACAGTCACTCGGCCGCTCAG-3′ PCR Template: BTG4-P412
BTG4-P412-Sp1-M2	F:5′-TTGAGGGGCGGGCGCGAGACGGGGC-3′ R:5′-CTCGGCCCGCGGGGTTCCAAGGACG-3′ PCR Template: BTG4-P412
BTG4-P412-Sp1-M3	F:5′-CTATGGGCGCGAGACGGGGCGGGTG-3′ R:5′-CGCCCCTCGGCCCGCGGGGTTCCAA-3′ PCR Template: BTG4-P412
BTG4-P412-Sp1-M4	F:5′-TTCGGGGATGGGGGGGGGGAGGAGG-3′ R:5′-CCCACCCGCCCCGTCTCGCGCCCGC-3′ PCR Template: BTG4-P412

The underlined sequences represent the restriction enzyme sites.

## Supplementary Materials

The following are available online at https://www.mdpi.com/2073-4425/11/2/217/s1, Figure S1: The constitutive and induction of BTG4 transcription is dependent on p53.
