# Supplementary material for: BTG4 is A Novel p53 Target Gene That Inhibits Cell Growth and Induces Apoptosis
Source: Genes (Basel). 2020 Feb 19;11(2):217. doi: 10.3390/genes11020217 (PMC7074044; doi:10.3390/genes11020217)
Supplement: Supplementary file 1 [file genes-11-00217-s001.pdf]

Supplementary Materials:

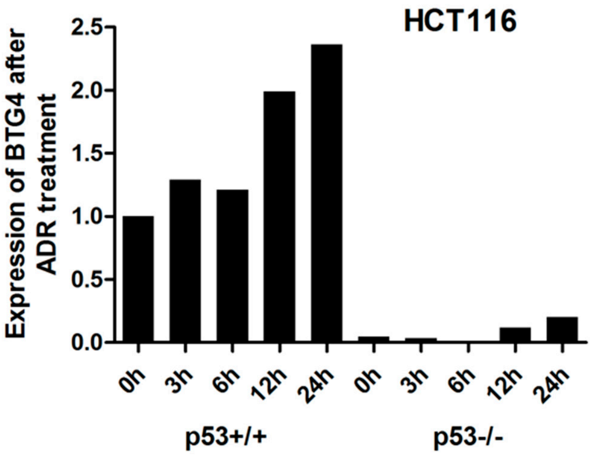

Supplementtary Figure S1. The constitutive and induction of BTG4 transcription is dependent on p53. HCT116 wild type and p53-/- cells were treated with adriamycin at the final concentration of 1μM. Cells were then collected and subjected to RT-PCR analysis at the indicated time points.
